# Clinical Characteristics and Surgical Management of Gastrointestinal Schwannomas

**DOI:** 10.1155/2020/9606807

**Published:** 2020-06-22

**Authors:** Xin Wu, Binglu Li, Chaoji Zheng, Xiaodong He

**Affiliations:** Department of General Surgery, Peking Union Medical College Hospital, Chinese Academy of Medical Sciences and Peking Union Medical College, Beijing, China

## Abstract

**Objectives:**

Schwannomas are tumors arising from Schwan cells of the neural sheath. Gastrointestinal schwannomas (GS) are rare and easily confused with a heterogeneous group of neuroectodermal or mesenchymal neoplasms. The aim of the present study is to analyze the clinicopathological features, surgical management methods, and long-term prognoses of GS patients.

**Methods:**

Between August 2004 and July 2019, 51 patients with GS were treated at the Peking Union Medical College Hospital. The medical records were reviewed retrospectively. A database containing demographic characteristics, clinical symptoms, imaging tests, operation details, pathological results, and prognoses was constructed and analyzed.

**Results:**

GS accounted for 2.0% of all schwannomas. The cohort comprised 19 men (37.3%) and 32 women (62.7%). The mean age was 55.7 ± 11.4 years. The most common symptom was abdominal pain (29.4%). Twenty-seven patients (52.9%) were asymptomatic and diagnosed incidentally. The most common tumor location of GS was the stomach (90.2%). S-100 had the highest positive rate (100%) in immunohistochemical staining. Forty-six patients (90.2%) were followed-up at a mean period of 49.5 ± 41.4 months. Forty-four patients (95.7%) survived without tumor, 1 patient survived with tumor, and 1 patient died. The 5-year cumulative overall survival rate and cumulative disease-free survival rate were 97.5% and 95.2%, respectively.

**Conclusion:**

GS are rare gastrointestinal tumors with favorable prognoses after surgical resection. Stomach is the most common site. Definitive diagnosis is determined by postoperative pathology. S-100 expression has diagnostic significance.

## 1. Introduction

Schwannomas are tumors arising from Schwan cells of the neural sheath [1]. They are most commonly found in the central nervous system, spinal cord, and peripheral nerves in the extremities [2]. Gastrointestinal schwannomas (GS), which originate from Auerbach's nerve plexus in the muscularis propria, are extremely rare [3, 4]. They were first described by Daimaru et al. in 1988 [5]. The most common site of GS is the stomach, followed by colon and rectum, and finally esophagus and small intestine [6]. GS are usually asymptomatic. A small number of symptomatic cases can present as bleeding, abdominal pain, a palpable mass, and changes in bowel habits [7, 8]. Because GS are rare and easily confused with a heterogeneous group of neuroectodermal or mesenchymal neoplasms such as gastrointestinal stromal tumors (GISTs), leiomyomas, neurofibromas, and glomus tumors, the preoperative diagnostic accuracy is quite low. Accurate diagnosis is established by postoperative pathology and immunohistochemistry [2, 6, 9].

Though GS are usually benign tumors and grow slowly, the recommended treatment is surgical resection. Endoscopic resection, laparoscopy, and open operation are all viable methods. The treatment choice depends on the tumor size and location and patients' willingness. There still are controversies regarding the clinical characteristics, perioperative management, histopathological features, and prognosis of GS. The purpose of the present study is to analyze the clinicopathological features, surgical management methods, and long-term prognoses of patients with GS, aiming at increasing the understanding of this rare disease among the medical community.

## 2. Materials and Methods

### 2.1. Patients

The medical records of patients with schwannomas who were treated at the Peking Union Medical College Hospital between August 2004 and July 2019 were reviewed retrospectively. The inclusion criteria were as follows: (1) patients who underwent surgery at our facility, (2) GS confirmed *via* postoperative pathology, (3) complete medical records. Patients with GISTs, leiomyomas, and other kinds of mesenchymal neoplasms were excluded. All the clinical data were collected from both inpatient and outpatient medical records and analyzed by 2 independent doctors. Postoperative complication was defined as any adverse event occurring within 30 days after operation. Telephone calls and outpatient interviews were used for follow-up. Computed tomography (CT) was performed every 6 months during the first year and annually thereafter, and endoscopy was performed annually. A discussion was held if there was discordance in the review work. A retrospective database containing the demographic characteristics, clinical symptoms, imaging tests, operation details, pathological results, and prognoses was constructed and analyzed.

### 2.2. Statistical Analysis

Statistical analysis was performed by an independent statistician. Linear variables are presented as mean ± standard deviation. Categorical variables are described using absolute number or frequency. Survival probability was estimated using the Kaplan-Meier method with log-rank test. All statistical analyses were conducted using Statistical Package for Social Sciences software (SPSS, version 25.0, IBM Corp., Armonk, NY, USA).

### 2.3. Ethics

The present study was approved by the Institutional Review Board of Peking Union Medical College Hospital (S-K962). All patients or their legal guardians provided written informed consent for the procedures performed. The need for informed consent for the publication of data was waived due to the retrospective nature of the study.

## 3. Results

A total of 2,529 patients with schwannomas were treated at Peking Union Medical College Hospital between August 2004 and July 2019. A total of 51 patients (2.0%) with GS were enrolled in this study according to the inclusion criteria. The demographic data and symptoms of the enrolled patients are presented in Table 1. GS were more common in females, and the male-to-female incidence ratio was 1 : 1.68. The most common symptom was abdominal pain, occurring in 15 patients (29.4%). Meanwhile, 27 patients (52.9%) were asymptomatic and diagnosed with a space-occupying lesion either incidentally or during routine health examination.

**Figure 1:**
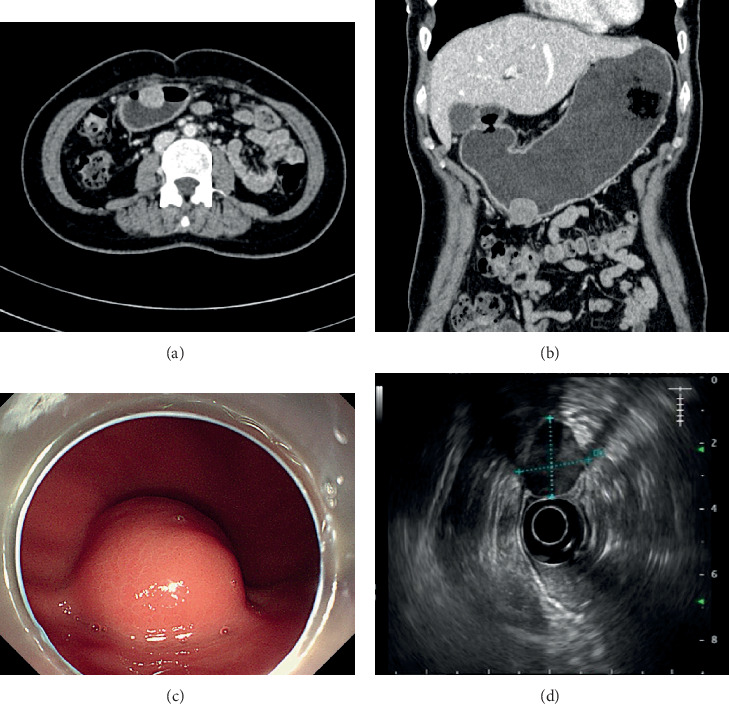
Characteristic findings of imaging modalities in patients with gastrointestinal schwannomas. (a, b) Computed tomography scan revealing a round, well defined, soft-tissue density tumor with moderate enhancement in axial plane (a) and coronal plane (b). (c) Endoscopy showing a domed submucosal lesion with intact overlying mucosa. (d) Endoscopic ultrasonography showing a hypoechoic mass on the gastric wall.

All patients underwent laboratory examinations including routine blood count, liver and kidney functions, and tumor marker screening. The results of the liver and kidney functions and routine blood count were unremarkable. However, CEA level was elevated in 1 patient (6.26 ng/ml; reference level, 0-5 ng/ml), and CA 19-9 level was elevated in 1 patient (34.7 U/mL; reference level, 0-34.0 U/mL). These two patients had no coexisting tumors. CT was performed in 39 patients and showed soft tissue density tumors that might have moderate enhancement. Gastrointestinal endoscopy was performed in 31 patients and revealed submucosal masses. Endoscopic ultrasonography was performed in 27 patients and showed muscularis propria space-occupying lesions. Characteristic findings of the diagnostic imaging are shown in Figure 1. Eight patients underwent endoscopic biopsies. Only 1 patient was diagnosed with spindle cell tumor preoperatively, while biopsy results of the other 7 patients showed inflammation.

All included patients received treatment at our hospital, which included laparoscopy (*n* = 30), laparotomy (*n* = 14), endoscopic resection (*n* = 4), and thoracotomy (*n* = 2). At the same time, 1 patient had undergone transanal rectal tumor resection at another hospital and was diagnosed with local recurrence and pelvic wall metastases 12 months later. Therefore, the patient only underwent transverse colostomy and rectal mass biopsy at our hospital. Postoperative complications occurred in 5 patients and included fever (*n* = 3), gastroplegia (*n* = 1), and ascites (*n* = 1). Fever was managed using antipyretics. Gastroplegia was treated with fasting, water deprivation, and parenteral nutrition. Ascites was cured by CT-guided percutaneous peritoneal drainage. In total, 3, 1, and 1 patients were classified as having grade I, grade II, and grade IIIa complications, respectively, following the Clavien-Dindo classification of surgical complications [10]. The postoperative morbidity rate was 9.8% (5/51). All patients were pathologically diagnosed with GS. Detailed information on the tumor location, treatment pattern, and tumor size is shown in Table 2. The most common tumor location for GS was the stomach (46/51, 90.2%), and the most used surgical procedure was laparoscopy (30/51, 58.8%). Immunohistochemical staining was performed for all patients, and the results are shown in Table 3. S-100 had the highest positive rate (100%) and was diagnostically significant. At the same time, 41 patients underwent Ki-67 index tests (median, 3%; range, 1%-60%).

As of November 2019, 46 patients (90.2%) were followed up at a mean period of 49.5 ± 41.4 months (range, 4 to 183 months), and 5 patients were lost to follow-up. Among the patients that were followed up, 44 patients (95.7%) survived without tumor recurrence. One patient with rectal schwannoma developed local recurrence and pelvic wall metastases 12 months after surgery, and she survived 38 months with tumor. Another patient with gastric schwannoma was found to have recurrence 6 months after surgery, and he died 12 months after surgery. Survival probability of the 46 followed up patients was estimated using the Kaplan-Meier method with log-rank test. The 5-year cumulative overall survival rate and cumulative disease-free survival rate were 97.5% and 95.2%, respectively.

## 4. Discussion

Gastrointestinal submucosal tumors are defined as intramural lesions underneath the mucosa and include a wide spectrum of benign-to-malignant neoplasms. They are divided into three major categories including GISTs, myogenic tumors, and neurogenic tumors [11, 12]. Schwannomas are a subset of neurogenic tumors and are the majority among them [12]. Schwannomas of the gastrointestinal tract are very rare and commonly occur in the stomach (approximately 0.2% of all gastric tumors) [13]. GS occur more frequently in female patients [14–16], with a female-to-male ratio of 2 : 1 or higher [17, 18]. In the present study, the female-to-male ratio was 1.68 : 1, which is slightly lower than that found in literature. GS are more common among the elderly, with the age of onset ranging from 50 to 60 years [14] and 41 to 60 years [17, 18]. In our series, the mean age of GS patients was 55.7 ± 11.4 years, which is consistent with the literature. Due to the indolent growth pattern, the majority of GS cases are asymptomatic [19, 20]. Some symptomatic patients may present with abdominal pain, gastrointestinal bleeding, or palpable mass [16]. The most common symptom is gastrointestinal bleeding followed by abdominal pain [21]. Most GS are discovered incidentally during routine health examination or on cross-sectional imaging. For gastric schwannomas, upper endoscopy is the most common method of discovery [3]. The results of this study also support the above findings; more than half of the patients were asymptomatic and diagnosed incidentally.

CT and endoscopy are the most valuable preoperative examinations for GS. GS are homogenous, well defined, round, and strongly contrast-enhanced tumors when visualized on CT scan [22]. In addition to the tumors, CT can also reveal the surrounding organs, which is very helpful for the operation. Although endoscopy can provide more information about the tumor location, nearly all mesenchymal tumors appear as smooth, round, submucosal masses with intact overlying mucosa [23]. In order to distinguish GS from other submucosal masses, endoscopic ultrasonography can be used. The possible features of GS are heterogeneous hypoechogenicity, a well-demarcated margin, and lack of cystic change [24]. However, GS with homogeneous hypoechoic internal echo pattern have also been reported in another study [6]. Because GS are submucosal, endoscopic biopsies usually show false-negative results. In our study, GS was diagnosed preoperatively in 0 out of 8 patients who underwent endoscopic biopsies. Endoscopic ultrasound-guided fine-needle aspiration can improve the preoperative diagnosis rate, with a diagnostic yield of 43% to 52% for all gastric submucosal tumors [25, 26]. One significant drawback of a biopsy is the theoretical risk of tumor spread and rupture, which would result in poor prognosis. We do not perform biopsy routinely at our hospital owing to this reason.

Surgical resection is the preferred treatment method in GS patients. It can prevent possible complications such as stenosis or bleeding [7]. In addition, the malignant potential of GS is another important factor that should be considered when determining treatment pattern. In most cases, tumor resection with negative margins is the standard of care, and no lymphadenectomy is recommended [7, 15]. When choosing surgical types, tumor location and size, the location of surrounding organs, as well as the operation skills of the surgeon are all important factors. As a result of new advances in gastroenterology, endoscopy plays an increasingly important role in the treatment of GS. Endoscopic full-thickness resection, endoscopic submucosal dissection, and submucosal tunneling endoscopic resection are all endoscopic modalities that provide less invasive ways to treat GS [27, 28]. In our series, 4 patients underwent endoscopic resection and all of them obtained negative surgical margins.

Only about 0.4% to 1% of all gastrointestinal submucosal tumors are GS, and a majority of them are in the stomach [15]. For gastric schwannomas, the most common lesion location is the gastric body (59.3%), followed by the antrum (26.7%), fundus (12%), and cardia (2%) [19, 29]. In the present study, gastric schwannomas accounted for 90.2% (46/51) of all GS. Gastric body accounted for 56.5% (26/46) of all gastric lesions, followed by antrum 34.8% (16/46), and fundus 8.7% (4/46). A definite diagnosis of GS is determined by paraffin sections and immunohistochemical examination. Schwannomas are uniformly positive for S-100, occasionally positive for CD34, and negative for CD117, desmin, and SMA [4, 21, 30–32]. In our series, the positive rates of S-100, CD34, desmin, SMA, CD117, and DOG-1 were 100%, 19.6%, 4.4%, 2.0%, 2.0%, and 0%, respectively. The results were consistent with the literature.

The main differential diagnosis for GS is GIST, which is much more common than GS. It is reported that for every 45 GISTs there is only 1 GS [18]. With similar presentation, and similar imaging and endoscopic results, preoperative differential diagnosis of GS is very difficult. However, since GISTs stain positively for CD34 and CD117 in paraffin sections, it is the gold standard method to distinguish between these two diseases. In addition, CT and endoscopy can also reveal some differences. GS more likely demonstrates smaller tumor size, round shape, and homogeneous enhancement pattern in CT than GIST. At the same time, perilesional lymph nodes and high vasculature are more frequently seen in GS, while cystic change is more common in GIST [33]. Unlike in other gastrointestinal tumors, perilesional lymph nodes are not a sign of malignancy in GS [34]. During endoscopic ultrasonography, GS present as heterogeneously hypoechoic lesions, which is helpful in differentiating them from other mesenchymal tumors [35].

GS usually have favorable prognosis. In the present study, the 5-year cumulative disease-free survival rate was over 95%. Incomplete surgical margins are the main cause of recurrence [6, 7]. Though malignant cases are extremely rare, they have been reported in several studies [36–38]. Because malignant GS are very difficult to distinguish from benign cases, even by postoperative histopathological examination, all patients should be followed-up regularly after operation.

This study has some limitations. First, because of its retrospective nature, the patient volume, registration information, treatment pattern, and variables assessed could not be designed beforehand. Second, due to the low incidence rate, the sample size was small. Third, gastric schwannomas accounted for most patients included, which may have resulted in some bias. Prospective, observational, and multicenter clinical trials are required to provide further supporting evidence with greater reliability.

## 5. Conclusions

GS are extremely rare gastrointestinal tumors with favorable prognoses after surgical resection. They account for only 2.0% of all schwannomas. The most common symptom is abdominal pain. Most patients are asymptomatic and diagnosed incidentally. Stomach is the most common site. Definitive diagnosis is determined by postoperative pathology. S-100 expression has diagnostic significance.

## Figures and Tables

**Table 1:** Demographic data and symptoms of patients with GS.

Characteristic	Value
Gender	*n* (%)
Male	19 (37.3%)
Female	32 (62.7%)
Age (years)	55.7 ± 11.4
Range	35-75
BMI (kg/m^2^)	23.6 ± 3.4
Symptoms	n (%)
Abdominal pain	15 (29.4%)
Melena	3 (5.9%)
Dysphagia	2 (3.9%)
Hematemesis	1 (2.0%)
Sour regurgitation	1 (2.0%)
Weight loss	1 (2.0%)
Chest pain	1 (2.0%)
None	27 (52.9%)

GS: gastrointestinal schwannomas; BMI: body mass index.

**Table 2:** Tumor location, treatment pattern, and tumor size of patients with GS.

Tumor location	n	Size (cm)	Laparoscopy (*n*)	Laparotomy (*n*)	ER (*n*)	TH (*n*)	TC (*n*)
Gastric body							
AW	12	3.5 ± 1.7	8	2	2		
PW	14	4.9 ± 2.9	9	5			
Gastric antrum							
AW	9	3.0 ± 1.1	5	3	1		
PW	7	3.6 ± 2.2	6	1			
Gastric fundus							
AW	2	2.9 ± 2.3	1			1	
PW	2	6.3 ± 1.1	1	1			
Esophagus	2	0.8 ± 0.8			1	1	
Duodenum	2	2.2 ± 0.9		2			
Rectum	1	10.3					1

GS: gastrointestinal schwannomas; ER: endoscopic resection; TH: thoracotomy; TC: transverse colostomy; AW: anterior wall; PW: posterior wall.

**Table 3:** Pathological immunohistochemistry results of patients with GS.

Items	Detected number (*n*)	Positive number (*n*)	Positive rate (%)
S-100	51	51	100
CD34	51	10	19.6
Desmin	45	2	4.4
SMA	51	1	2.0
CD117	50	1	2.0
DOG-1	45	0	0

GS: gastrointestinal schwannomas.

## Data Availability

The data used to support the findings of this study are included within the article.
